# Supplementary material for: Rapid Detection of Thermal Treatment of Honey by Chemometrics-Assisted FTIR Spectroscopy
Source: Foods. 2021 Nov 22;10(11):2892. doi: 10.3390/foods10112892 (PMC8623053; doi:10.3390/foods10112892)
Supplement: Supplementary file 1 [file foods-10-02892-s001.zip › Supplementary tables.pdf]

Table S1

HMF content in honey after heat treatment, measured by chromatography.

Sample	Average HMF content (mg/kg)	SD (mg/kg)	RSD (%)
Acacia RAW	9.43	0.53	5.57
Acacia 70°C, 15 min	8.48	0.23	2.56
Acacia 70°C, 120 min	10.6	0.03	0.30
Acacia 40°C, 3.5 h	12.85	0.33	2.57
Acacia 40°C, 24 h	10.00	0.38	3.82
Eucalyptus RAW	26.51	0.87	3.29
Eucalyptus 70°C, 15 min	28.33	0.38	1.37
Eucalyptus 70°C, 120 min	28.73	0.39	1.37
Eucalyptus 40°C, 3.5 h	34.39	0.47	1.38
Eucalyptus 40°C, 24 h	37.26	0.45	1.21
Orange RAW	16.10	0.64	4.025
Orange 70°C, 15 min	16.31	0.021	0.13
Orange 70°C, 120 min	8.65	0.11	1.28
Orange 40°C, 3.5 h	16.19	0.21	1.34
Orange 40°C, 24 h	16.43	0.22	1.34

SD - standard deviation, RSD - relative standard deviation

Table S2

Fructose, glucose, and sucrose concentrations in different types of honey, measured by chromatography.

Sample	Fructose concentration ± SD (%)	Glucose Concentration ± SD (%)	Sucrose Concentration ±SD (%)
Acacia RAW	32.45 ± 0.14	20.91 ± 3.025	0.33 ± 0.0031
Acacia 70°C, 15 min	36.47 ± 0.057	21.03 ± 0.003	0.39 ± 0.015
Acacia 70°C, 120 min	37.64 ± 0.084	21.67 ± 0.011	0.35 ± 0.017

Acacia 40°C, 3.5 h	38.37 ± 0.105	22.28 ±0.019	0.32 ± 0.0011
Acacia 40°C, 24 h	38.26 ± 0.16	22.61 ± 0.603	0.32 ± 0.0061
Eucalyptus RAW	38.54 ± 1.19	21.33 ± 0.66	0.14 ± 0.012
Eucalyptus 70°C, 15 min	33.39 ± 0.051	29.57 ± 3.71	0.13 ± 0.0091
Eucalyptus 70°C, 120 min	36.07 ± 3.51	27.11 ± 0.61	0.12 ± 0.0044
Eucalyptus 40°C, 3.5 h	35.90 ± 0.01	23.45 ± 0.014	0.13 ± 0.0098
Eucalyptus 40°C, 24 h	36.12 ± 0.016	24.00 ± 0.012	0.14 ± 0.0052
Orange RAW	34.36 ± 0.019	24.71 ± 0.62	4.078 ± 0.018
Orange 70°C, 15 min	29.95 ± 0.011	22.30 ± 0.002	3.55 ± 0.195
Orange 70°C, 120 min	35.28 ± 0.029	26.03 ± 0.02	4.32 ± 0.0052
Orange 40°C, 3.5 h	35.30 ± 0.012	26.15 ± 0.021	4.43 ± 0.012
Orange 40°C; 24 h	32.72 ± 0.011	23.99 ± 0.59	3.80 ± 0.015

Table S3. Accuracy and precision of honey classification after heat treatment.

Sample	Overall accuracy	Average precision
Acacia honey heated up to 40°C	0.815	0.824
Acacia honey heated up to 70°C	0.868	0.877
Eucalyptus honey heated up to 40°C	0.895	0.922
Eucalyptus honey heated up to 70°C	0.947	0.958
Orange blossom honey heated up to 40°C	0.873	0.864
Orange blossom honey heated up to 70°C	0.922	0.917
