# Supplementary figures and images for: Rapid Detection of Thermal Treatment of Honey by Chemometrics-Assisted FTIR Spectroscopy
Source: Foods. 2021 Nov 22;10(11):2892. doi: 10.3390/foods10112892 (PMC8623053; doi:10.3390/foods10112892)

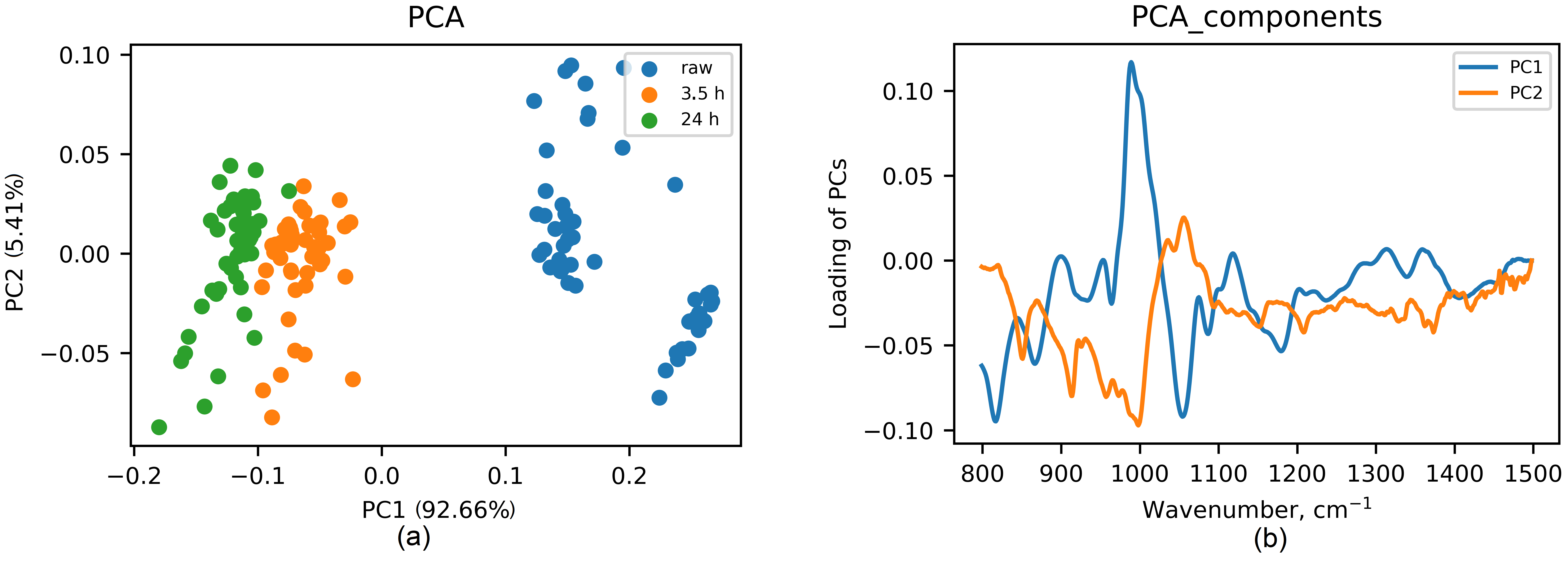

Supplement: Supplementary file 1 [file foods-10-02892-s001.zip › FigS3.tif]

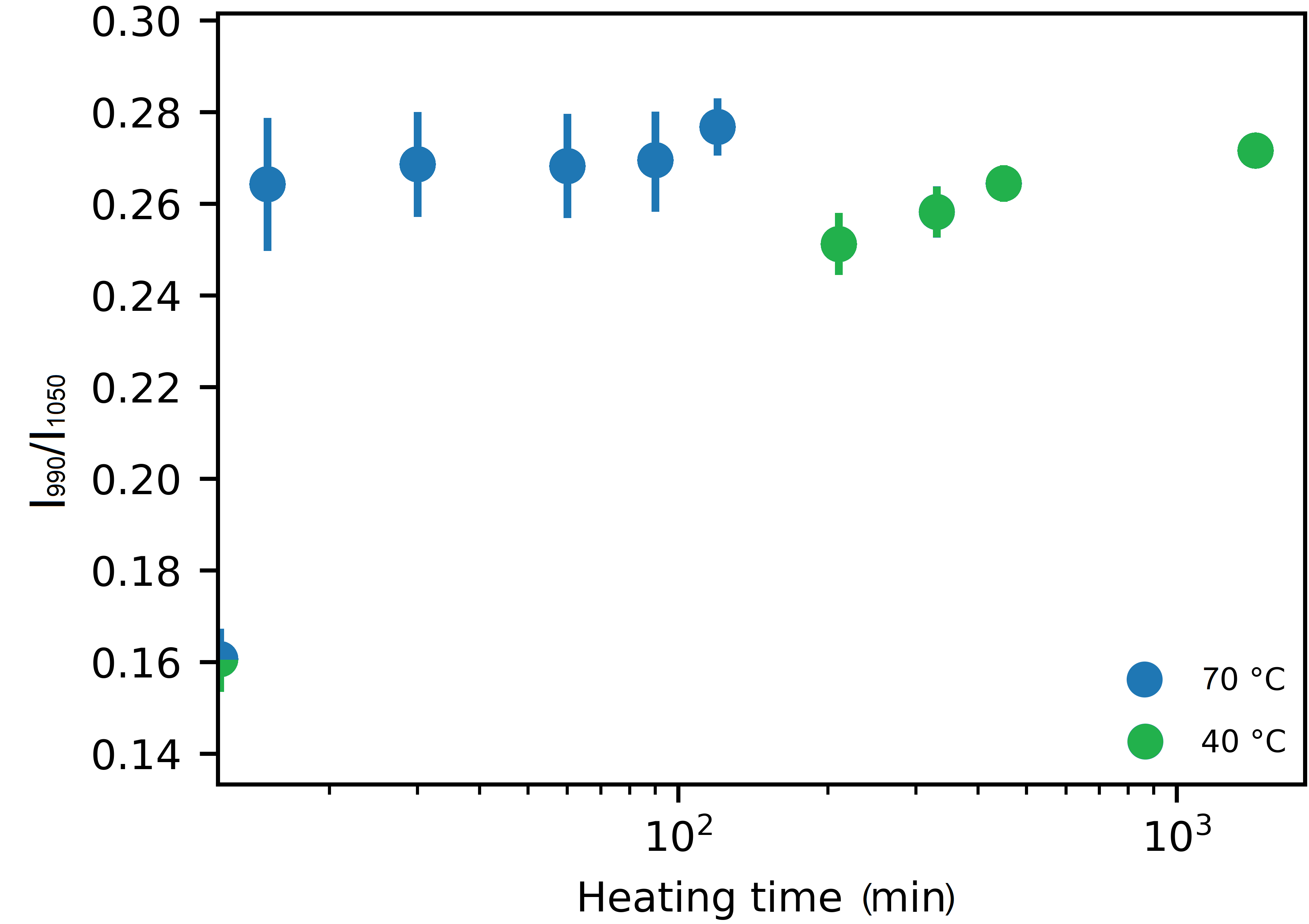

Supplement: Supplementary file 1 [file foods-10-02892-s001.zip › FigS4.tif]

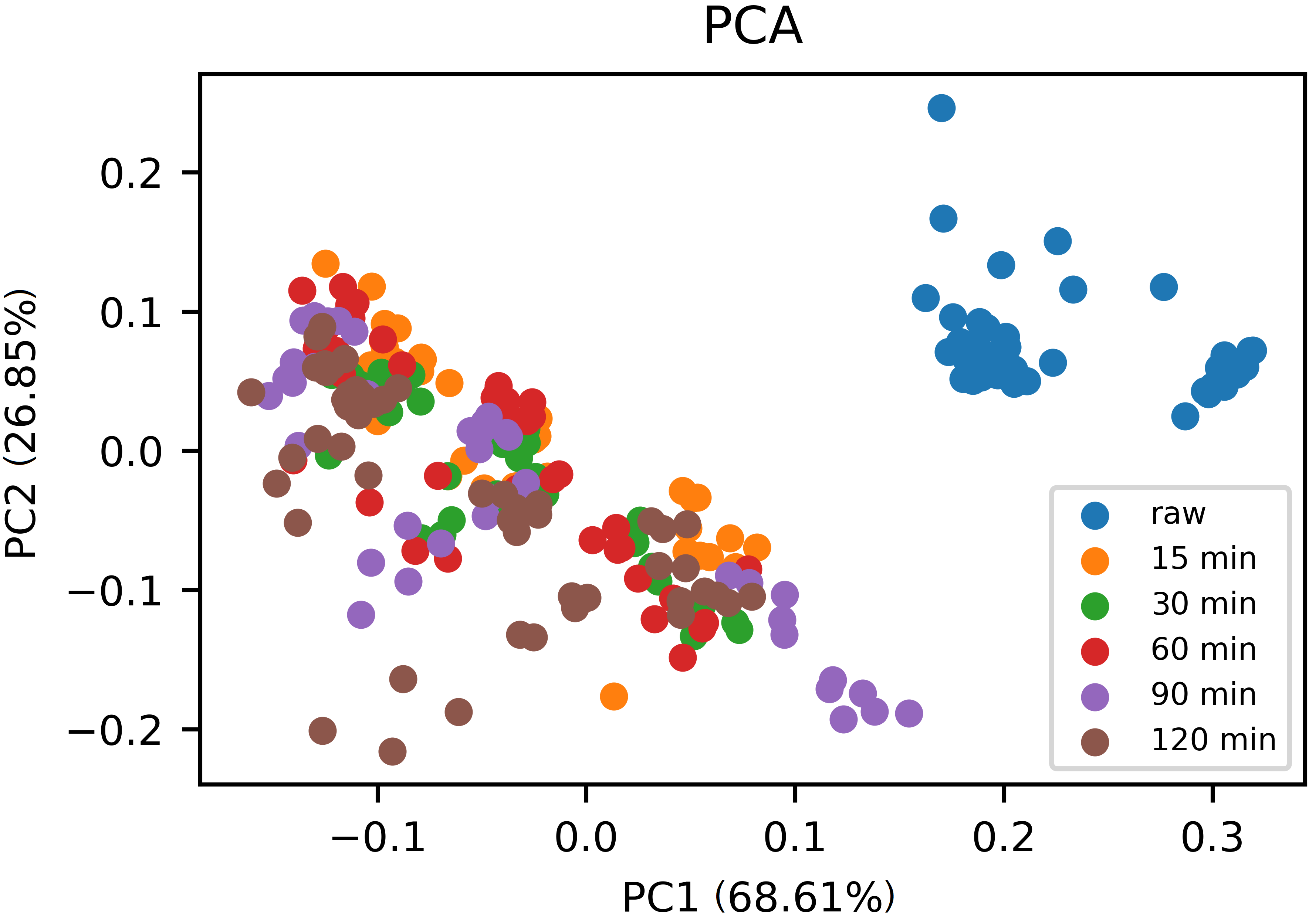

Supplement: Supplementary file 1 [file foods-10-02892-s001.zip › FigS1.tif]

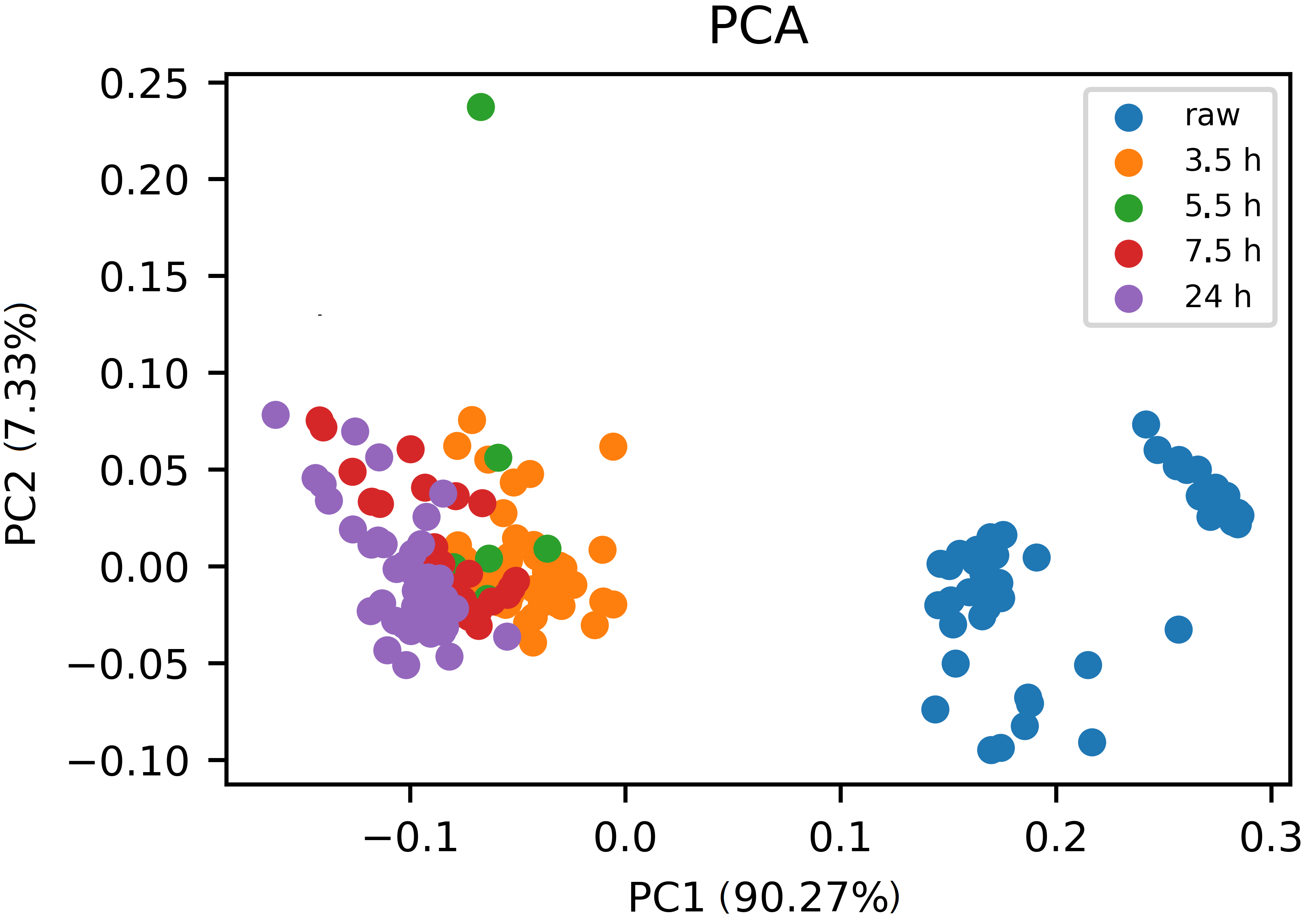

Supplement: Supplementary file 1 [file foods-10-02892-s001.zip › FigS2.tif]
